# *Trypanosoma cruzi* TcSMUG L-surface Mucins Promote Development and Infectivity in the Triatomine Vector *Rhodnius prolixus*

**DOI:** 10.1371/journal.pntd.0002552

**Published:** 2013-11-14

**Authors:** Marcelo S. Gonzalez, Marcela S. Souza, Eloi S. Garcia, Nadir F. S. Nogueira, Cícero B. Mello, Gaspar E. Cánepa, Santiago Bertotti, Ignacio M. Durante, Patrícia Azambuja, Carlos A. Buscaglia

**Affiliations:** 1 Laboratório de Biologia de Insetos, Departamento de Biologia Geral, Instituto de Biologia, Universidade Federal Fluminense, Morro do Valonguinho S/N, Centro, Niterói, Rio de Janeiro, Brazil; 2 Instituto Nacional de Entomologia Molecular (INCT-EM, CNPq), Brazil; 3 Laboratório de Bioquímica e Fisiologia de Insetos, Instituto Oswaldo Cruz, Fiocruz, Rio de Janeiro, Brazil; 4 Laboratório de Biologia Celular e Tecidual, Centro de Biociências e Biotecnologia, Universidade Estadual do Norte Fluminense, Horto, Campos dos Goytacases, Rio de Janeiro, Brazil; 5 Instituto de Investigaciones Biotecnológicas-Instituto Tecnológico de Chascomus (IIB- INTECH), Universidad Nacional de San Martín (UNSAM) - Consejo Nacional de Investigaciones Científicas y Técnicas (CONICET), Instituto de Investigaciones Biotecnológicas “Dr Rodolfo Ugalde”, Campus UNSAM, San Martín (1650), Buenos Aires, Argentina; National Institutes of Health, United States of America

## Abstract

**Background:**

*TcSMUG L* products were recently identified as novel mucin-type glycoconjugates restricted to the surface of insect-dwelling epimastigote forms of *Trypanosoma cruzi*, the etiological agent of Chagas disease. The remarkable conservation of their predicted mature N-terminal region, which is exposed to the extracellular milieu, suggests that TcSMUG L products may be involved in structural and/or functional aspects of the interaction with the insect vector.

**Methodology and Principal Findings:**

Here, we investigated the putative roles of TcSMUG L mucins in both *in vivo* development and *ex vivo* attachment of epimastigotes to the luminal surface of the digestive tract of *Rhodnius prolixus*. Our results indicate that the exogenous addition of TcSMUG L N-terminal peptide, but not control *T. cruzi* mucin peptides, to the infected bloodmeal inhibited the development of parasites in *R. prolixus* in a dose-dependent manner. Pre-incubation of insect midguts with the TcSMUG L peptide impaired the *ex vivo* attachment of epimastigotes to the luminal surface epithelium, likely by competing out TcSMUG L binding sites on the luminal surface of the posterior midgut, as revealed by fluorescence microscopy.

**Conclusion and Significance:**

Together, these observations indicate that TcSMUG L mucins are a determinant of both adhesion of *T. cruzi* epimastigotes to the posterior midgut epithelial cells of the triatomine, and the infection of the insect vector, *R. prolixus*.

## Author Summary

Chagas disease, the major tropical human disease in much of Latin America, affects approximately 11 million people. There are 300,000 new cases of Chagas disease and approximately 21,000 deaths, annually. Triatomine vectors, including *Rhodnius prolixus*, are able to transmit the protozoan *Trypanosoma cruzi*, the etiological agent of disease. To develop within insects, the flagellates undergo morphological changes, modulating surface molecules to enable interactions with insect tissues such as the perimicrovilar membranes in the midgut which is an essential step for their development and successful transmission to a vertebrate host. The surface of *T. cruzi* is covered in glycosyl phosphatidylinositol (GPI)-anchored mucin molecules that determine parasite protection and establishment of a persistent infection in vertebrates. A particular kind of mucin, termed TcSMUG L, is only present at surface of the insect-dwelling stages of protozoan and, according to our results, it is involved in the interaction between *T. cruzi* and its invertebrate host, determining both the *ex vivo* adhesion to the insect midgut cells and the *in vivo* development in the vector. Collectively, our work adds new insight into the relevance of mucin-type glycoconjugates in the infection of insect vectors and points to them as promising targets to develop transmission-blocking strategies for this disease.

## Introduction

Described by its discoverer, Carlos Chagas [1], [2], as “one of the most injurious tropical illnesses, specially to children in contaminated areas, either in determining a chronic sickly condition in which people become unable to perform vital activities or as an important factor of human degeneration,” Chagas disease remains a major tropical human disease in much of Latin America, affecting approximately 11 million people. There are 300,000 new cases of Chagas disease each year, with approximately 21,000 deaths annually [3]. Various triatomine vectors, including *Rhodnius*, *Triatoma* and *Pastrongylus*, are able to acquire and transmit *Trypanosoma cruzi*, the etiological agent of Chagas disease [4], [5]. During their development within insects, parasites undergo profound morphological changes, modulating surface molecules to enable interactions with specific insect tissues that are essential for their survival, development and successful transmission to a vertebrate host [6], [7]. *T. cruzi*-insect vector interactions begin when the insect feeds on the blood of an infected vertebrate host. Once ingested, most of the bloodstream trypomastigotes differentiate into non-infective epimastigote forms. In the posterior midgut, they repeatedly divide by binary fission and adhere to perimicrovillar membranes (PMM) secreted by the underlying midgut epithelial cells [8]–[11]. In the rectum, a proportion of epimastigotes attaches to the rectal cuticle through hydrophobic interactions and transforms into non-replicative infective metacyclic trypomastigotes, which are released together with insect feces and urine during blood feeding [12]–[14].

The entire surface of *T. cruzi* is covered in glycosylphosphatidylinositol (GPI)-anchored mucin molecules that determine parasite protection and establishment of a persistent infection in vertebrate hosts [15]. *T. cruzi* mucins comprise a large gene family that can be split into two major groups, termed *T. cruzi* mucin gene family (*TcMUC*) and *T. cruzi* small mucin-like gene family (*TcSMUG*), based on sequence comparisons [16]. *TcMUC* codes for more than 1,000 polymorphic products, which are largely co-expressed on the surface of the mammal-dwelling stages [16]–[18]. In addition to their putative immune modulatory role [17], [19], one particular *TcMUC* product termed TSSA (trypomastigote small surface antigen) was recently shown to be involved in trypomastigote adhesion to non-macrophagic cells [20]. The second mucin group, *TcSMUG*, displays significantly less diversity and codes for very small open reading frames. Upon processing of the signal peptide and GPI-anchoring signal, the average predicted molecular mass for the mature apo-mucins would be ∼7 kDa, with Thr representing as much as 50% of the residues. The hydroxyl groups of some of these Thr residues are further derivatized with short *O*-linked oligosaccharide chains in the Golgi/post-Golgi compartments, which increases the molecular mass of the mature mucins to 35–50 kDa, depending on both the particular *TcSMUG* product and the parasite isolate [21], [22]. *TcSMUG* is composed of two subgroups of genes, named *L* and *S*, which display >80% identity on average. Mass spectrometry analyses identified *TcSMUG S* products as the backbone for the 35/50 kDa mucins (known as Gp35/50 mucins) expressed on the surface of insect-dwelling stages [22]. Upon transmission to the mammalian host, Gp35/50 mucins on the surface of metacyclic trypomastigotes bind to non-macrophagic cells in a receptor-mediated manner and induce a bidirectional Ca^2+^ response, which likely contributes to host-cell invasion [15]. Recent data indicated that *TcSMUG L* products, though not revealed in the *T. cruzi* proteomic data sets published so far, constitute a novel mucin-type glycoconjugate restricted to epimastigote forms [22]–[26]. In addition to displaying substantial structural homologies and a common evolutionary origin, comparative analyses highlighted certain differences between *TcSMUG L* and *TcSMUG S* products [26]. First, *TcSMUG L* products, unlike those of *TcSMUG S*, are not acceptors of sialic acid residues, likely due to the absence of terminal β-Gal residues in the proper configuration. Secondly, and at variance with *TcSMUG S* products that are expressed at fairly similar levels on every *T. cruzi* stock, *TcSMUG L* expression seems quite variable among different parasite isolates. Finally, the remarkable conservation of *TcSMUG L* deduced products within the predicted mature N-terminal peptide, which does not undergo *O*-glycosylation, suggest that they are under positive selection against diversification [26]. Because of these features, it has been speculated that structural and/or functional constraints rather than immunological issues limit *TcSMUG* diversification.

In the present work, we investigated the role of TcSMUG L mucins in the attachment of *T. cruzi* epimastigotes from the Dm28c stock to the midgut epithelium of *R. prolixus* and the consequent development of the protozoan in the insect vector.

## Materials and Methods

### Insects and Parasites

*R. prolixus* (Hemiptera: Reduviidae) were obtained from a longstanding colony reared in the laboratory at 28°C and 60–70% relative humidity [27] where they were fed on chickens weekly and raised as previously described [28]. For the *in vivo* experiments, the insects were fasted for approximately 15 days and were then fed with infected heat-inactivated citrated human blood using an artificial apparatus similar to that described previously [29]. The *T. cruzi* Dm28c clone, classified in the TcI phylogenetic group [30], was maintained in Novy-MacNeal-Nicolle media (NNN) and brain heart infusion media (BHI- DIFCO) supplemented with bovine serum albumin (BSA) and hemin. For the *in vivo* and *ex vivo* experiments, epimastigotes were collected during the exponential growth phase, washed three times in 0.15 M NaCl, 0.01 M phosphate-buffer, pH 7.2 (PBS) and used immediately [11], [31].

### Ethics Statement

*R. prolixus* were fed and raised according to the Ethical Principles in Animal Experimentation approved by the Ethics Committee in Animal Experimentation (CEUA/FIOCRUZ) under the approved protocol number P-54/10-4/LW12/11. The experiments performed with citrated human blood using an artificial apparatus were conducted according to the Ethical Principles in Animal Experimentation approved by the Ethics Committee in Animal Experimentation (CEUA/FIOCRUZ) under the approved protocol number L-0061/08. All blood donors provided informed written consent. Both protocols are from CONCEA/MCT (http://www.cobea.org.br/), which is associated with the American Association for Animal Science (AAAS), the Federation of European Laboratory Animal Science Associations (FELASA), the International Council for Animal Science (ICLAS) and the Association for Assessment and Accreditation of Laboratory Animal Care International (AAALAC).

### Mucin Purification

Epimastigotes (10^9^) were delipidated using a water/chloroform/butan-1-ol treatment and further extracted with butan-1-ol at 4°C as described previously [32]. Briefly, the soluble fraction was evaporated under an N_2_ stream, and the insoluble material was re-extracted with 66% butan-1-ol in water. The butan-1-ol phase (F1) contained mainly lipids, phospholipids and glycoinositolphosphates (GIPLs), whereas the aqueous phase (F2) is enriched in mucins [32]. Both phases were further extracted with 9% butan-1-ol in water. Delipidated parasite pellets were also extracted with 9% butan-1-ol in water and the mucin-rich aqueous (F3) and butan-1-ol (F4) phases were stored. The final parasite pellets were resuspended in denaturing loading buffer containing 6 M urea and 100 µg/ml DNAse I (SIGMA).

### Concanavalin A (ConA)-Fractionation and Phosphatidilinositol-Specific Phospholipase C (PI-PLC) Treatment

In order to enrich in glycoconjugates, pellets containing 10^8^ parasites were homogenized in ConA buffer (50 mM Tris-HCl, pH 7.4, 150 mM NaCl, 1% NP40, 0.1% Na deoxycholate, 1 mM PMSF, 50 µM TLCK, 1 mM DTT) and fractionated in batch using 200 µl of ConA-sepharose (GE Healthcare) [26]. Elution was carried out with 300 µl of ConA buffer with 0.5 M α methylmannoside (Sigma, St. Louis, MO). Parasite total lysates were treated with PI-PLC and submitted to Triton X-114 partition as described [26], to ascertain the presence of GPI anchor.

### Gel Electrophoresis and Western Blots

Gel electrophoresis was performed under denaturing conditions in 15% SDS-PAGE. For Western blots using total proteins, lysates corresponding to ∼10^7^ parasites prepared as described [26] were loaded in each lane, transferred to PVDF membranes (GE Healthcare), reacted with the appropriate antiserum followed by HRP-conjugated secondary Abs (Sigma) and developed using chemiluminescence (Pierce). Antibodies to TcSMUG L were affinity-purified and used as described by [26]. Rabbit antiserum to glutamate dehydrogenase from *T. cruzi* (TcGDH) was used at 1∶3,000 dilution [33].

### Peptides

Peptides used in this study were synthesized bearing an acetyl group on their N-termini and a C-terminal Cys residue (GenScript). Sequences were derived from the predicted N-terminal region of mature *TcSMUG L* (AVFKAAGGDPKKNTTC), *TcSMUG S* (VEAGEGQDQTC) and *TSSA* (TPPSGTENKPATGEAPSQPGAC) products. When indicated, peptides were synthesized with a biotin group instead of the acetyl group on their N-termini. Although bioinformatics methods indicate that the sequences EEGQYDAAVFAVFKAAGGDPKKNTT and EEGQYDAAVFVEAGEGQDQT constitute the predicted mature N-termini for TcSMUG L and S products, respectively [26], mass spectrometry-based data using purified epimastigote total mucins [34], strongly suggested a further trimming of the EEGQYDAAVF sequence *in vivo*.

### *Ex Vivo* Interaction between *R. prolixus* Posterior Midgut Cells and *T. cruzi* Epimastigotes

After washing in PBS, epimastigotes were suspended in fresh BHI to a density of 2.5×10^7^ cells/ml. Samples of an interaction medium composed of 200 µl of this parasite suspension together with posterior midguts, freshly dissected and washed only in PBS, from insects collected 10 days after a non-infectious blood meal, were placed in Eppendorf microtubes [10] and incubated for 30 min at 25°C (non-treated control group). Under these conditions, epimastigotes adhered to the luminal surface of midgut epithelium cells [11]. For the experimental groups, the midguts were previously incubated (30 min, 25°C) in PBS supplemented with TcSMUG S (negative control), TcSMUG L or TSSA peptides at different concentrations. The treated-posterior midguts were then washed in fresh PBS and immediately added to the BHI interaction medium containing parasites. After incubation (30 min, 25°C), all midgut preparations were spread onto glass slides to count the number of attached parasites. A Zeiss microscope with reticulated ocular, equipped with a video microscopy camera, was used for counting parasites attached to 100 randomly chosen epithelial cells in 10 different fields of each midgut preparation. For each experimental group, 10 insect midguts were used [35], [36].

### *In Vivo* Infection Assays

Fifth-instar nymphs of regularly fed *R. prolixus*, which had been starved for 7 days after the last ecdysis, were fed on artificial bloodmeal apparatus with a mixture of heat-inactivated citrated human blood and epimastigotes (2×10^5^ parasites/ml) as previously described [37]. TcSMUG S (negative control), TSSA or TcSMUG L peptide was added to the infected blood meal to a final concentration of 30 µg/ml just before feeding. At days 7, 14 or 21, the entire digestive tracts consisting of anterior midgut (stomach), posterior midgut and rectum of 10 insects were dissected and homogenized in a small volume of PBS. Afterwards, additional PBS was added to fill the homogenates to 1 ml [38], [39]. The number of parasites in each homogenate was determined using a Neubauer hemocytometer [40], [41]. Each experiment was repeated at least three times.

### Light Microscopy

Posterior midgut compartments obtained by dissection were fixed for 2 h at room temperature in 2.5% glutaraldehyde diluted in 0.1 M cacodylate buffer, pH 7.2, and washed twice in the same buffer. Post-fixation was performed in the dark for 2 h in 1% osmium tetroxide diluted in 0.1 M cacodylate buffer, pH 7.2, followed by dehydration with continuous acetone series (70%, 90% and 100%, respectively). Samples were then embedded in epoxy resin and polymerized at 60°C for three days. Thick plastic sections were stained with toluidine blue and observed under an Axioplan MC 100 spot microscope [10].

### Fluorescence Microscopy and Histochemical Studies

Dissected posterior midgut fragments were fixed for 1 h at room temperature in 4% *p*-formaldehyde diluted in 0.1 M cacodylate buffer, pH 7.2. Afterwards, samples were washed in PBS containing 1% of BSA, pH 7.2 (PBS-BSA) and incubated for 30 min in 50 mM ammonium chloride solution followed by another washing step in PBS-BSA at room temperature. Tissues were then incubated with biotin-labeled TcSMUG S, TSSA or TcSMUG L peptide diluted in PBS-BSA for 1 h at room temperature and washed again in PBS-BSA before incubation with FITC-labeled-Avidin conjugate (SIGMA) (1∶100) for 1 h and washed in distilled water in the dark for 10 min [42]. For the control groups, the incubation with biotin-labeled peptides was omitted. Finally, the tissues were spread onto glass slides for visualization using an emission filter of 488 nm and observed under an Axioplan MC 100 spot microscope coupled to an Axiovision system computer [43].

### Data Analysis

Results were analyzed using ANOVA and Tukey's tests [44] using Stats Direct Statistical Software, version 2.2.7 (StatsDirect Ltd., Sale, Cheshire, UK). Differences between treated- and control-groups were considered non-statistically significant when *p*>0.05. Probability values are specified in the text.

## Results

### *TcSMUG L* Products Are Expressed as Mucin-Like Molecules in Dm28c Epimastigotes

**Figure 1:**
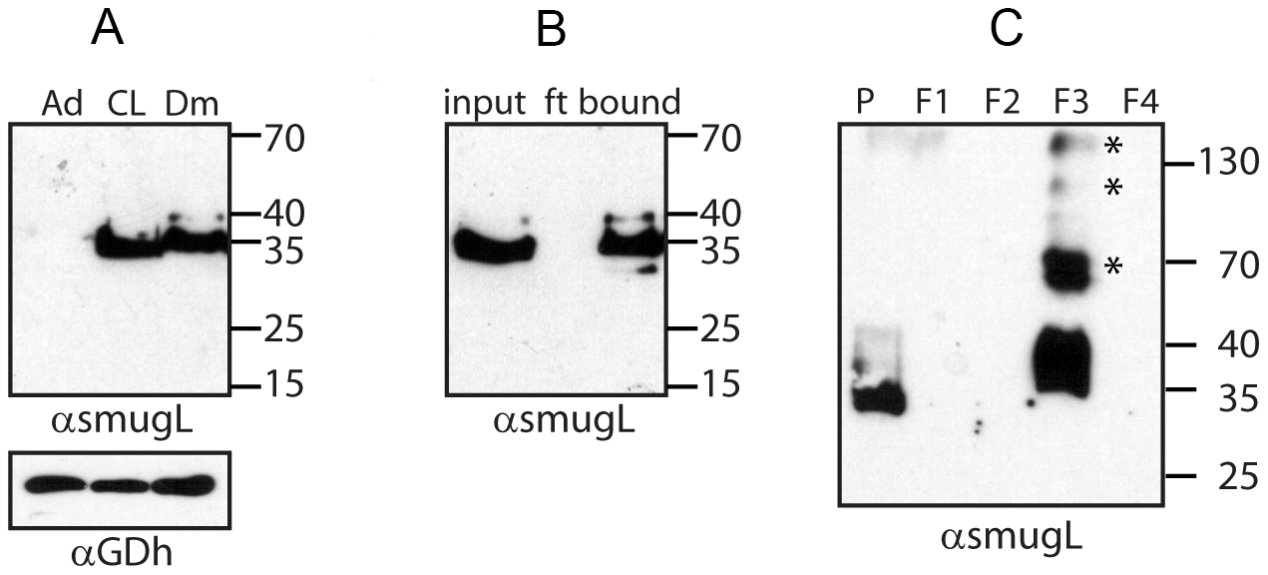
Western blots of *TcSMUG L* products from *T. cruzi*. A) Extracts of epimastigotes from different parasite stocks (Ad, Adriana; CL, CL Brener; Dm, Dm28c) were probed with either anti-TcSMUG L antibodies or anti-glutamate dehydrogenase (GDH) antiserum. B) ConA-fractionated extracts of Dm28c epimastigotes were probed with anti-TcSMUG L antiserum. ft, flow-through. C) Butan-1-ol extraction analysis of Dm28c delipidated epimastigotes. Fractions, named according to [19], were probed with affinity-purified anti-TcSMUG L antibodies. Molecular mass markers (in kDa) are indicated at right. *Denotes aggregates.

Previous results indicate that the expression level of *TcSMUG L*-encoded products is quite variable among epimastigotes from different *T. cruzi* isolates [26]. Therefore, as a first step toward the validation of our *R. prolixus* infection model, we undertook preliminary characterization of *TcSMUG L* products in the DM28c stock. Western blotting assays carried out over total epimastigote lysates and probed with affinity-purified antibodies directed against an N-terminus-derived TcSMUG L peptide revealed a major ∼35 kDa band, thus in the range of fully processed *TcSMUG L* products described in other parasite stocks [26] (Fig. 1A). As controls, we used analogous fractions from epimastigotes from Adriana and CL Brener stocks, which showed the greatest differences in terms of *TcSMUG L* expression [26]. The results were normalized by re-probing the membrane with antiserum directed against TcGDH. Densitometric analyses indicated that *TcSMUG L* expression levels from the DM28c stock were roughly equivalent (86%) to that of CL Brener. These products were removed from the parasite surface following PI-PLC treatment [26], a molecular signature of GPI-anchored molecules (not shown), and were specifically retained following ConA chromatography (Fig. 1B), indicating they bear terminal α-D-mannosyl and/or α-D-glucosyl residues, as described for other stocks [26]. To analyze whether *TcSMUG L* products behaved as mucin-type proteins, *i.e.*, underwent extensive *O*-glycosylation, we purified total mucins from Dm28c epimastigotes following a standard butan-1-ol extraction protocol [32] and probed these fractions by Western blot. As shown in Fig. 1C, *TcSMUG L* products were mostly detected in the F3 fraction, which was highly enriched in gp35/50, as verified by mAb 2B10 and 10D8 reactivity (not shown). The presence of high-molecular weight aggregates in purified *TcSMUG L* products has been described for other *T. cruzi* mucin-type glycoconjugates [22], [26]. A minor fraction was also revealed in the pellet, which might be ascribed to incomplete extraction. Together, these results strongly suggest that Dm28c epimastigotes express high levels of fully processed *TcSMUG L* product on their surface.

### *TcSMUG L* Products Are Involved in Epimastigote *Ex Vivo* Attachment to *R. prolixus* Posterior Midgut Epithelium

**Figure 2:**
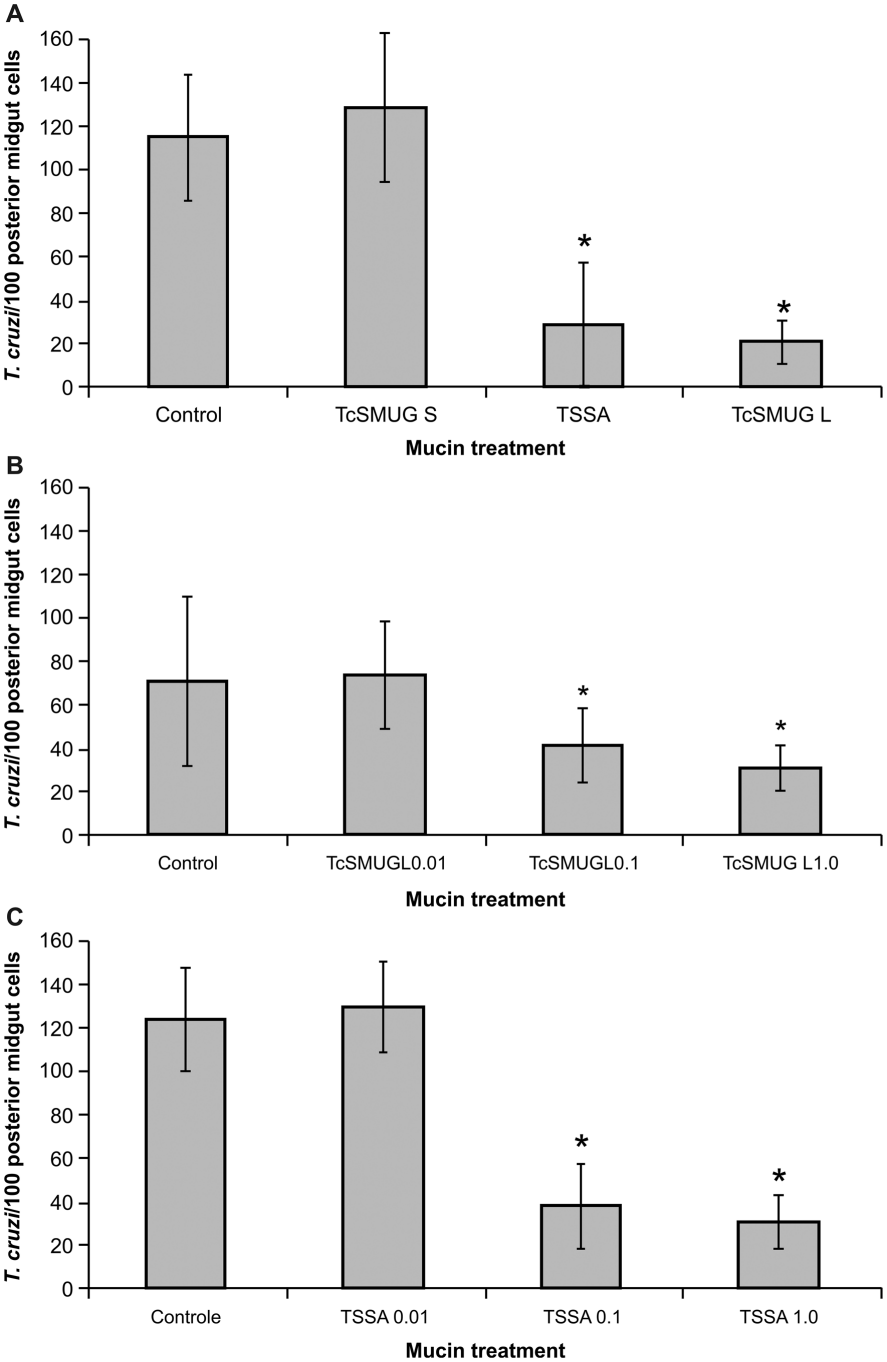
Effect of surface mucins on *ex vivo T. cruzi* attachment to the midgut epithelium of *Rhodnius prolixus*. Midguts obtained from male fifth-instar nymphs 10 days after the bloodmeal were previously incubated for 30 min in PBS supplemented with the indicated mucin peptides and added with BHI interaction medium containing flagellates (2.5×10^7^/ml). Pre-incubation with mucin peptides was omitted in control (non-treated) group. Adhered epimastigotes were counted per 100 epithelial cells in 10 different fields of each midgut preparation. (A) Pre-incubation in 1 µg/ml of TcSMUG S, TSSA or TcSMUG L. (B) Pre-incubation in 0.01, 0.1 or 1.0 µg/ml of TcSMUG L. (C) Pre-incubation in 0.01, 0.1 or 1.0 µg/ml of TSSA. Each group represents mean ± S.D. of parasites attached in 10 midguts. Asterisk represents experimental groups with statistical significance compared to the control. *Trypanosoma cruzi* small mucin S (TcSMUG S), *Trypanosoma cruzi* small mucin L (TcSMUG L) and trypomastigote small surface antigen (TSSA).

To assess whether *TcSMUG L* products can act as direct ligands for possible receptors in insect epithelial midgut cells, we tested the effect of pre-treatment of dissected midguts with a peptide spanning the *TcSMUG L* mature N-terminus. As controls, we assayed in parallel the effect of the corresponding peptide derived from *TcSMUG S* and *TSSA*, a member of the *TcMUC* family of mucins. As a first set of experiments, in posterior *R. prolixus* midgut preparations obtained from a control (non-treated) group, 114.8±28.2 epimastigotes were found attached per 100 midgut cells (Fig. 2A). Similar adhesion rates (128.8±34.7/100 midgut cells) were obtained when midguts were first incubated with 1 µg/ml of a control TcSMUG S peptide (p>0·05) (Fig. 2A). In contrast, attachment of only 28.5±28.4 and 20.8±10.06 epimastigotes per 100 cells of the midgut epithelium were recorded when the flagellates were pre-incubated with 1 µg/ml of either TcSMUG L or a control TcMUC-derived (TSSA) peptide (p<0·0001), respectively (Fig. 2A). A dose-dependent effect on the *ex vivo* attachment of epimastigotes was verified for the latter molecules, indicating that the presence of either synthetic peptide blocked a potential ligand-receptor interaction involved in epimastigote attachment (Fig. 2B). As shown in Fig. 2B, incubation with 0.01 µg/ml of the TcSMUG L peptide did not affect flagellate adhesion rates when compared with the control group, whereas incubation with 0.1 µg/ml or 1 µg/ml of the TcSMUG L peptide reduced *T. cruzi* attachment to 40.8 ±16.78 and 30.8 ±10.42 (p<0·01) epimastigotes per 100 midgut cells, respectively. Similarly, midgut incubation with 0.01 µg/ml of the TSSA peptide resulted in 128.6±20.87epimastigotes attached per 100 midgut cells and did not affect flagellate adhesion rates when compared with the control group (123.2±23.74 epimastigotes/100 midgut cells), whereas incubation with 0.1 µg/ml or 1 µg/ml of the same peptide reduced *T. cruzi* attachment to 37.6 ±19.65 and 30.6 ±12.4 (p<0·001) epimastigotes per 100 midgut cells, respectively (Fig. 2C). Therefore, our results showed that the pre-incubation of *R. prolixus* midguts with the TcSMUG L or TSSA peptide promote significant alteration of the epimastigote-midgut interaction rate.

### *TcSMUG L* Products Are Involved in *T. cruzi In Vivo* Development in the Insect Vector

**Figure 3:**
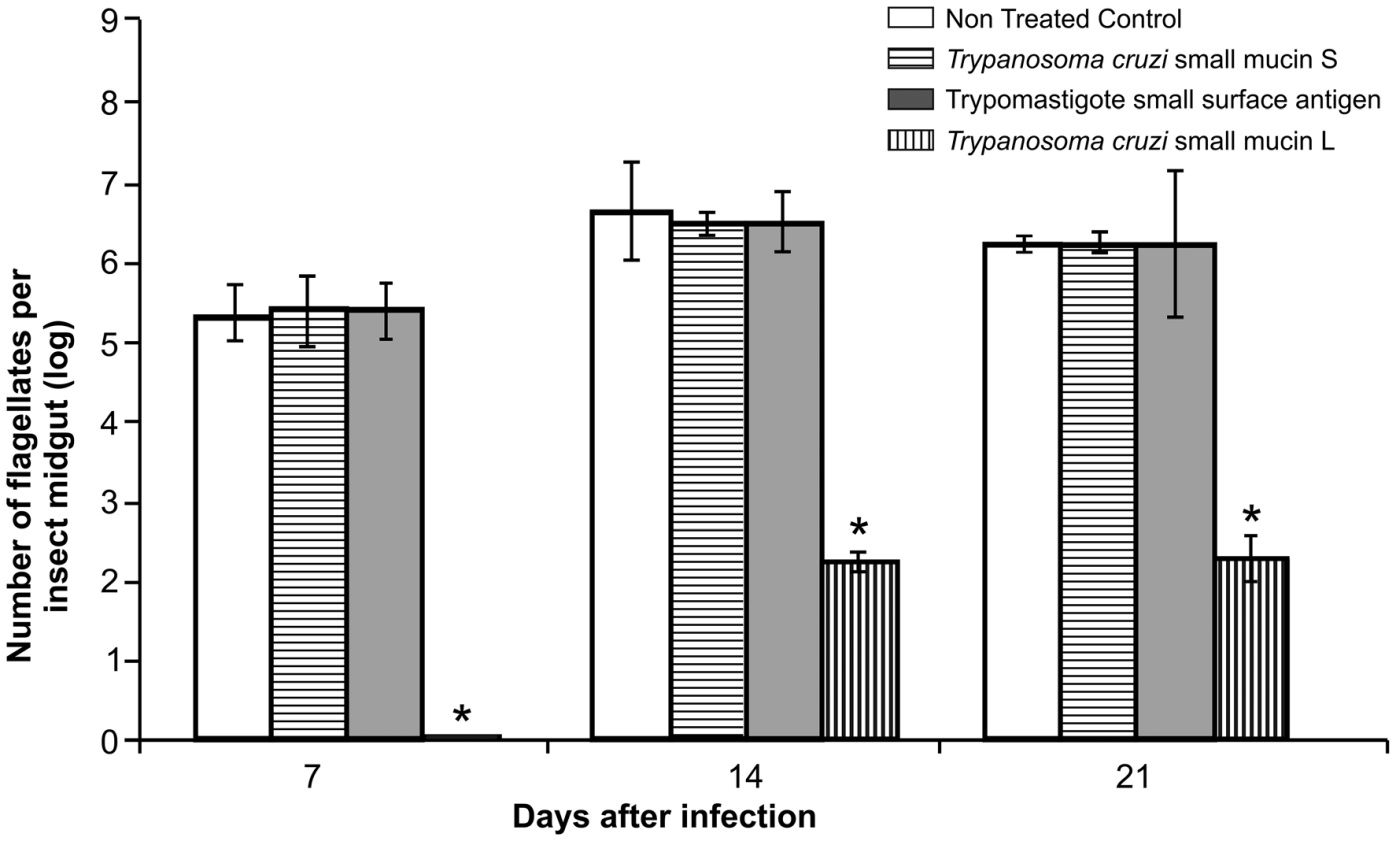
Effect of surface mucins on *T. cruzi in vivo* development in the digestive tract of *Rhodnius prolixus*. Insects were fed on citrated, complement-inactivated human blood containing 2×10^5^ flagellates/ml. Each mucin peptide was added to the bloodmeal at a concentration of 30 µg/ml and insects dissected as days 7, 14 or 21 post feeding. Each point represents mean±S.D of flagellates/ml in the whole gut of 10 insects. Asterisk represents experimental groups with statistical significance compared to the control.

Upon ingestion of approximately 2×10^5^ Dm28c epimastigotes/ml of blood, fifth-instar nymphs of *R. prolixus* became heavily infected with *T. cruzi* (Fig. 3). In the control group, the infection levels varied from 3.33±0.35×10^5^ flagellates/ml of digestive tract homogenate 7 days after infection to 2.06±0.10×10^6^ flagellates/ml of digestive tract 21 days post-infection. Similar infection levels were observed throughout the time frame of the experiment in insect groups fed with blood supplemented with either TcSMUG S or TSSA peptide (p>0·05). In contrast, nymphs fed with blood supplemented with TcSMUG L peptide showed significantly reduced infection levels. Direct counts revealed 2.3±0.12×10^2^ (p<0·0001) and 2.3±0.27×10^2^ (p<0·0001) flagellates/ml of digestive tract homogenate 14 and 21 days post-infection, respectively, representing a ∼4-log difference from controls. Even more compelling, no parasites were observed 7 days post-infection in TcSMUG L peptide-treated insects. Together, these results suggest that soluble TcSMUG L peptide significantly inhibits the normal development of Dm28c parasites in *R. prolixus*, likely by interfering between the interaction of endogenous *TcSMUG L* products displayed on the surface of epimastigotes and triatomid midgut receptors.

### Light Microscopy and Histochemical Localization of TcSMUG L Recognition Sites in the Posterior Midgut of *R. prolixus*

**Figure 4:**
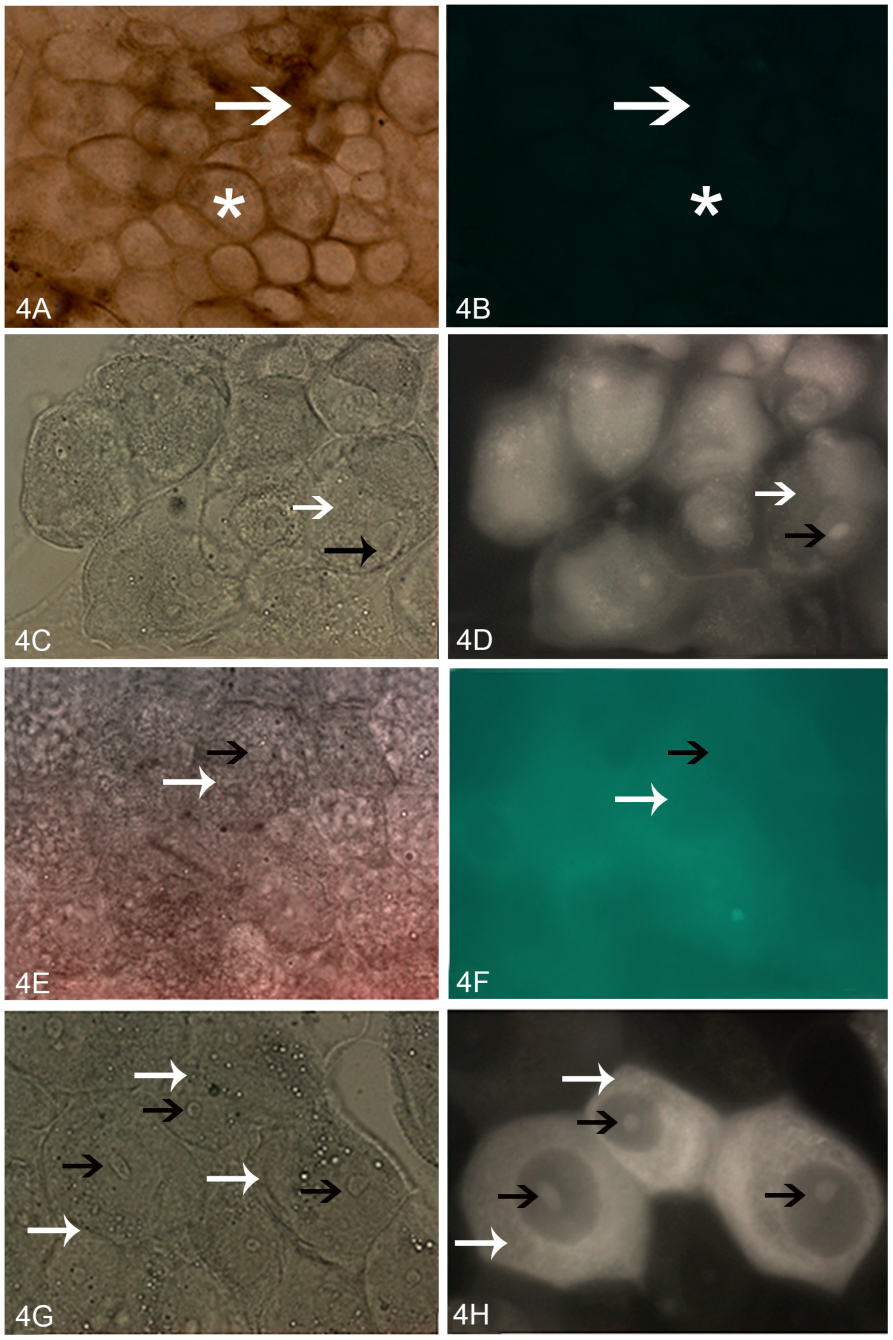
Photomicrographs of posterior midgut epithelial cells of fifth-instar *R. prolixus* incubated with biotin-labeled peptides. (A) Light microscopy showing single-globe columnar epithelial cells(white star) and PMM (white arrow). (B) Fluorescence microscopy showing that no demarcation was observed after incubation with avidin-FITC-labeled conjugate alone. Light and fluorescence microscopy, respectively, of samples incubated with biotin-labeled TcSMUG L (C and D), biotin-labeled TcSMUG S (E and F), and biotin-labeled TSSA (G and H). Fluorescence of the surface and nucleolus of the midgut cells is indicated by white and black arrows (respectively). 400×.

Light microscopy of *R. prolixus* midgut showed a single columnar epithelium composed by posterior midgut cells. Toluidine-stained granules were observed in the apical and medial region, where a round nucleus was located. As previously described [10], these epithelial cells were closely joined at their medial and basal regions, whereas a brush border associated with the PMM was observed at the luminal surface of their apical regions (Fig. S1). No significant labeling was obtained after incubation of *R. prolixus* posterior midgut surface with Avidin-FITC conjugate alone (Fig. 4A, B) or after previous incubation with biotin-labeled TcSMUG S peptide followed by the Avidin-FITC conjugate (Fig. 4E, F). However, in line with previous results, fluorescence of specific binding sites was observed on the surface of luminal posterior midgut cells after pre-incubation with biotin-labeled TcSMUG L (Fig. 4C, D) or TSSA (Fig. 4G, H) peptide under the same conditions. Unexpectedly, the samples pre-incubated with TSSA also showed some intracellular staining, particularly in the nucleolus, which may be attributed to partial permeabilization of the cells during fixation.

## Discussion

During its life cycle, *T. cruzi* adheres to specific host molecules/cell types as essential steps for parasite survival. Depending on the parasite developmental stage and the nature of the involved molecules, these interactions trigger a variety of events such as bidirectional cell signaling, host cell internalization, parasite replication or transformation to infective stages [45], [46]. Within the triatomid vector, different lines of research have established that molecules able to inhibit parasite attachment to insect tissues *ex vivo* also often efficiently block the *in vivo* development of *T. cruzi* [35]. For instance, purified GIPLs were shown to bind to the luminal surface of the posterior midgut. Accordingly, their exogenous addition dramatically impaired both *ex vivo* attachment of epimastigotes to this organ and the flagellate multiplication in the insect digestive tract, which prevented the successful colonization of the vector [11]. Similar effects were described for different carbohydrate-binding proteins (CBPs) of the epimastigote surface with a strong affinity for higher glycan oligomers and sulfated glycosaminoglycans (S-GAGs) present in the posterior midgut of *R. prolixus* [36], [47], [48]. The net negative charge of both S-GAGs and specific carbohydrates may act as a first, non-specific step prior to *T. cruzi* adhesion to specific receptors in the luminal midgut PMM [35]. In addition, an antiserum raised against *R. prolixus* PMM and midgut tissue interfered with midgut structural organization and slowed the development of *T. cruzi* in the insect vector [49].

The entire surface, including the cell body and the flagellum, of various *T. cruzi* developmental forms is covered with mucins that play a key role in parasite protection [50]–[52], infectivity, and development [15]. *T. cruzi* mucins are anchored to the outer leaflet of the plasma membrane through a GPI motif and undergo extensive glycosylation in their central Thr-rich domain. These features confer strong hydrophilic characteristics and an extended (“rod-like”) structural conformation [53], which is often used to elevate an outermost peptide above the parasite glycocalix. This N-terminal peptide, which is not predicted to be *O*-glycosylated, is thus ideally suited to participate in cell-to-cell interaction phenomena [54].

The results presented here strongly suggest that the N-terminal peptide of *TcSMUG L* products is required for efficient interaction between the parasite and the insect midgut and the subsequent growth of the flagellate in the invertebrate host. As shown, addition of the exogenous peptide led to a significant reduction in *ex vivo* adhesion to the insect midgut, and also inhibition of *in vivo* development within vectors. Due to its small molecular size, this effect is unlikely to be caused by steric effects, where the TcSMUG L peptide would prevent access of parasite recognition molecules to specific sites in the insect gut cells. Quite the opposite, we favor the hypothesis that the exogenous TcSMUG L peptide exerts its inhibitory effect by outcompeting the parasite binding sites in the triatomine luminal surface of the midgut epithelium. This idea is further supported by histochemical data showing intense labeling of the surface of luminal posterior midgut cells after pre-incubation with biotin-labeled TcSMUG L peptide. Therefore, it is likely that *TcSMUG L* products act as surface adhesion molecules, promoting epimastigote adhesion and colonization through recognition of specific receptor(s) on insect cells. In this framework, a distinct expression profile verified for *TcSMUG L* products [26] could contribute to the biological heterogeneity found between different isolates of *T. cruzi* in terms of triatomid infectivity. Moreover, drastic reduction in *TcSMUG L* expression upon differentiation to metacyclic trypomastigotes suggests a developmental regulation program that could help to explain why these latter forms are detached from the midgut surface [26].

One unexpected and puzzling finding was that the exogenous TSSA-derived peptide showed adhesion properties to insect midgut cells, as well as *ex vivo* inhibition on epimastigote attachment. It is worth mentioning that *TSSA* belongs to the *TcMUC* group of genes, which is expressed during the mammalian-dwelling stages of the protozoan [20], [21], [54]. In particular, *TSSA* expression is restricted to the surface of blood trypomastigotes, the parasite stage ingested by the vector during an infective blood meal, and amastigote-to-bloodstream trypomastigote intermediate forms. From a structural staindpoint, and despite showing similar bias in amino acid composition (with Cys, Phe, Trp and Tyr amino acids -all residues that could perturb the physicochemical properties of *T. cruzi* mucins- being underrepresented or absent), there are no obvious similarities in the primary sequences of the TSSA and TcSMUG L peptides that could explain their similar binding properties. Indeed, the labeling pattern obtained for TSSA in posterior midgut sections is different than that obtained for the TcSMUG L peptide, suggesting they recognize different receptor(s) on the surface of insect cells, although more studies would be required to address this point. Importantly, and in strict correlation with its expression profiling, the interaction between TSSA and insect midgut cells seems to have no biological relevance, as it had no effect on parasite *in vivo* development.

Although little is known about the mechanisms leading to the remodeling of the surface coat when the flagellate moves from the mammal into the insect vector, it is reasonable to suppose that TSSA is shed during this process. Free in the insect stomach, TSSA may reach the posterior midgut and be recognized by PMM receptors for mucins or other glycoconjugates. Transfer of antigenic epitopes from *T. cruzi* to the PMM of *Triatoma infestans* has been previously described [55]. In spite of this, TSSA does not seem to participate in the protozoan development of *R. prolixus*, which is compatible with its lack of expression in insect-dwelling stages of *T. cruzi*.

Altogether, these findings establish that *TcSMUG L* products are involved in the interaction between *T. cruzi* and its invertebrate host. Indeed, our results demonstrate that these products are involved in successful adhesion to the epithelial cells of insect vectors both *ex vivo* and *in vivo*, although the exact molecular mechanism, and particularly the putative receptor on the surface of the insect cells, should be further explored. Most importantly, a severe reduction in flagellate population in the digestive tract of *R. prolixus* was observed when triatomines were infected with epimastigotes of *T. cruzi* and simultaneously orally treated with the TcSMUG L peptide. Collectively, our work adds new insight into the relevance of mucin-type glycoconjugates in the infection of insect vectors and points to them as promising targets to develop transmission-blocking strategies for this disease.

## Supporting Information

Figure S1 **Light microscopy of toluidin blue-stained posterior midgut cells of** ***R. prolixus*** **10 days after feeding.** Oblique (a) and transverse (b) sections of the apical region of columnar epithelial cells, with brush border associated with perimicrovillar membranes (thick black arrow), round nuclei (thin black arrow) and the posterior midgut lumen (L). 400×.
(TIF)
