# Supplementary figures and images for: Trypanosoma cruzi TcSMUG L-surface Mucins Promote Development and Infectivity in the Triatomine Vector Rhodnius prolixus
Source: PLoS Negl Trop Dis. 2013 Nov 14;7(11):e2552. doi: 10.1371/journal.pntd.0002552 (PMC3828161; doi:10.1371/journal.pntd.0002552)

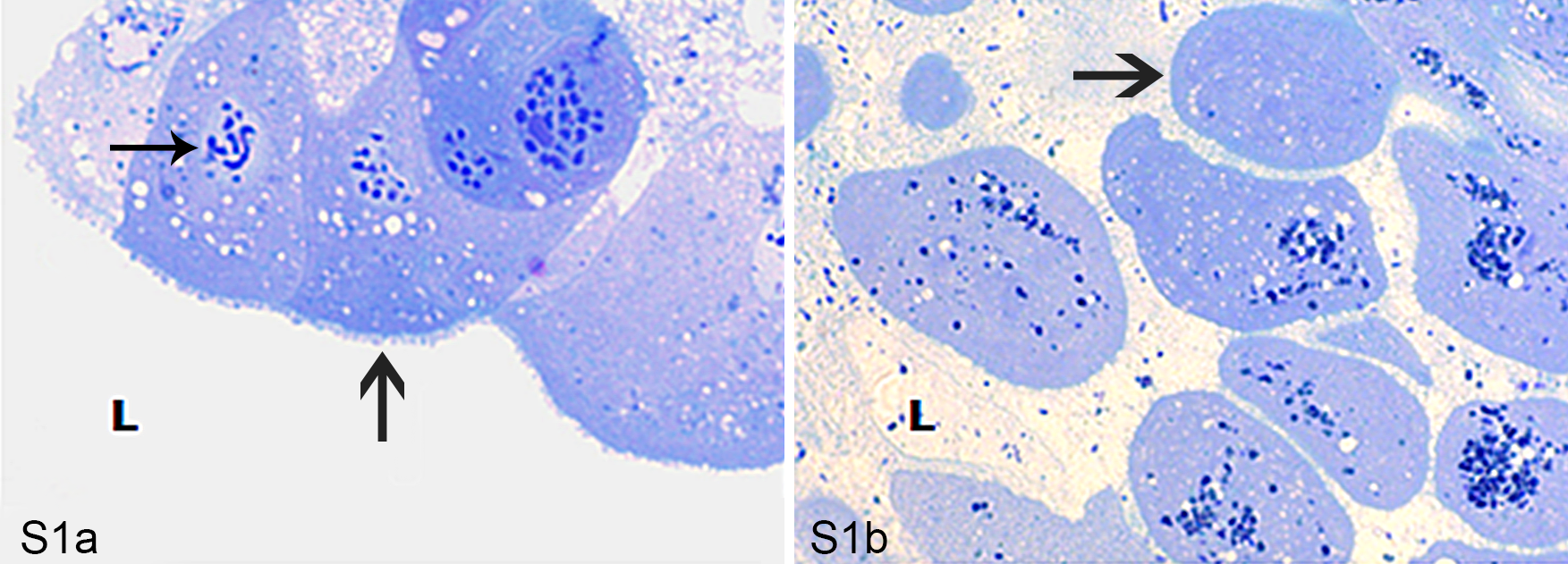

Supplement: Figure S1 — Light microscopy of toluidin blue-stained posterior midgut cells of R. prolixus 10 days after feeding. Oblique (a) and transverse (b) sections of the apical region of columnar epithelial cells, with brush border associated with perimicrovillar membranes (thick black arrow), round nuclei (thin black arrow) and the posterior midgut lumen (L). 400×. (TIF) [file pntd.0002552.s001.tif]
